# Surgical Approach to a Large Left Adrenocortical Mass with Associated Tumour Thrombosis of the Left Renal Vein: Preservation of the Ipsilateral Kidney

**DOI:** 10.1155/2009/365805

**Published:** 2009-12-13

**Authors:** Manuel Pérez Utrilla, Carlos Nuñez Mora, Alejandro Rojo Sebastián, Pedro M. Cabrera Castillo, José M. García Mediero

**Affiliations:** ^1^Urology Department, MD Anderson International Spain, Hospital General Ciudad Real, 28050 Madrid, Spain; ^2^Pathology Department, MD Anderson International Spain, 28050 Madrid, Spain

## Abstract

A sixty-years-old male with diagnosis of a left adrenal mass (146 × 99 × 126 mm) with associated tumour thrombosis of the left renal vein with no clear signs of thrombosis of the inferior vena cava was admitted for elective surgery Finally an adrenalectomy and excision of tumour thrombus preserving the ipsilateral kidney was made. Despite of the complex vascular management, this kind of approaches allow to preserve normal renal function in patients with future nephrotoxic treatment like cisplatin.

## 1. Introduction

Primary adrenocortical carcinoma is a tumour of low incidence and poor prognosis. Between 0.05% and 0.2% of cancer deaths are due to this tumour [1]. 

Its poor prognosis is due mainly to the absence of effective adjuvant treatment and the delay in diagnosis, caused by two factors. In first place, its anatomical location allows it to reach a considerable size without causing symptoms. Secondly, approximately 50% of large suprarenal masses are functioning, causing Cushing's syndrome in most cases while the remainder is diagnosed incidentally [2]. 

Although the prognostic factors are unclear, various studies indicate that surgical resection of the mass may improve survival rates and even in those advanced cases in which complete oncological resection is not possible, and it may help to improve the results of chemotherapy [3].

## 2. Case Report

Sixty-year old male with no medical history of interest was referred to the Urology Department with the incidental finding of a left renal mass and space-occupying lesions (SOL) in the liver. Physical examination revealed a mobile, slightly painful mass in the left hypochondrium. Laboratory tests found high levels of alkaline phosphatase (350 IU/l), GGT (115 IU/l), and LDH (883 IU/l). Analytical studies showed normal adrenal hormones in blood and urine.

**Figure 1:**
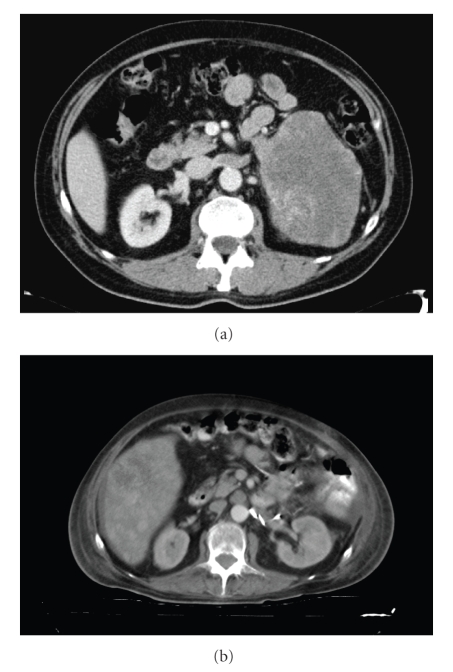
Pre- (a) and postoperative (b) CT scan showing tumour thrombosis of the left renal vein and later vascular result.

Abdominal ultrasound revealed a left renal mass of heterogeneous echogenicity. Multiple liver SOLs were also identified (most about 6 cm in diameter) in the right liver lobe and an image suggestive of a cyst in the head of the pancreas. An axial tomography was performed (thorax-abdominal-pelvic slices) with intravenous contrast which showed the presence of multiple pulmonary nodules, some larger than 1 cm in diameter, suggestive of metastases, and Left suprarenal mass, 146 × 99 × 126 mm, suggestive of malignancy, with no separation from the kidney and which displaced the pancreas and spleen with no clear signs of infiltration. Tumour thrombosis involves suprarenal vein and left renal vein with no clear signs of thrombosis of the inferior vena cava (IVC) (Figure 1). 

He was admitted for elective surgery with a diagnosis of left adrenocortical carcinoma with thrombosis of left renal vein and liver and lung metastases. A bilateral subcostal incision was made. Mobilisation of left colon and splenorenal ligaments expose a large left suprarenal mass with renal vein thrombosis and significant tumour adhesions. After dissection of the renal hilum (double artery and single vein), the suprarenal vein was then dissected. The renal vein was clamped at vena cava ostium, the renal hilum was clamped en bloc, and venotomy was performed in the proximal third. Complete dissection and enucleation of the tumour thrombus, closure with Prolene 5/0 and unclamping, check the haemostasia. The left suprarenal vein was then tied and the surgical specimen was excised, preserving the ipsilateral kidney. Finally, regional lymphadenectomy was performed.

**Figure 2:**
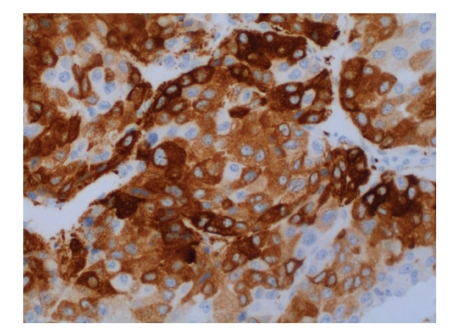
Inhibin expression (immunohistochemistry); origin marker in adrenal cortex.

**Figure 3:**
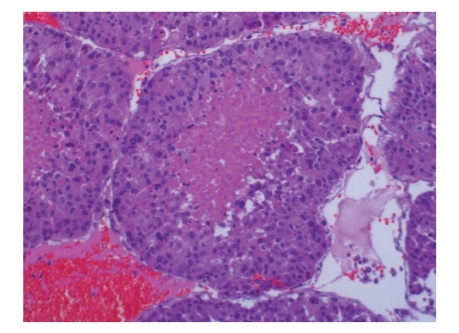
Papillar fragment of embolized tumour with necrotic axis and viable tumoral surface.

The anatomical pathology diagnosis was *adrenocortical carcinoma* measuring 15 cm × 6 cm × 11 cm (in its longitudinal, transverse, and anteroposterior diameters) with *tumour embolism in the left renal vein*, measuring 4 cm × 2 cm × 1 cm (L × T × AP). None of the adenopathies were infiltrated, a total of 9 having been excised (Figures 2 and 3).

The patient was discharged 6 days after surgery, with normal creatinine levels and no complications. He is presently on treatment with mitotane in increasing doses awaiting to associate treatment with adriamycin and cisplatin.

## 3. Discussion

Adrenocortical carcinoma may have renal vein tumour thrombosis, ranging from 9% to 19% of cases [4]. It is precisely in these cases when we have to consider the indication of ipsilateral nephrectomy combined with adrenalectomy and excision of the tumour thrombus. According to literature [5–7], of the 121 published cases of adrenalectomies with associated tumour thrombosis (AATT), 71.2% of cases were primary adrenocortical carcinomas, 15.8% were phaeochromocytomas, 5.5% were neuroblastomas, 2.5% were leiomyosarcomas, and 5% were metastases of diverse origin (transitional cell carcinoma of the renal pelvis, small cell lung cancer, thyroid cancer, and Wilms' tumour). 

Massive involvement of the inferior vena cava or infiltration of the venous wall itself may be considered limiting factors for the surgical indication, also bearing in mind the poor prognosis of adrenocortical cancer and the technical difficulties that adequate vascular control and its subsequent reconstruction entail. For this reason, the preoperative study of the tumour thrombosis and the involvement of the renal vein and/or inferior vena cava has become particularly important. Computerised axial tomography (CT) and magnetic resonance (MR) are considered two imaging techniques which are of most value in vascular study, taking into account that MR also offers the advantage of properly distinguishing between a fibrinogenic and a tumour thrombus [8].

The approach should therefore be planned considering factors such as the size of the suprarenal mass, the upper limit of the tumour venous thrombosis, and the need for procedures associated with the adrenalectomy, such as nephrectomy, splenectomy, regional lymphadenectomy, and others [7]. Our team prefers to preserve the ipsilateral kidney in these cases, provided that it has no local infiltration. This is not always technically possible, and hence, in the study by Lucon et al. [6], the 8 AATT (3 phaeochromocytomas and 5 adrenocortical carcinomas) was combined with ipsilateral nephrectomy. Similarly, Chiche et al. [7] carried out a literature review and presented their experience with 15 AATT. They combined ipsilateral nephrectomy in 10 cases. Another nephrectomy was subsequently performed due to ischaemia after excision of the thrombus, highlighting the complex vascular approach in these types of interventions when attempting to preserve the renal function. In the anatomical pathology findings, 8 of the 11 renal specimens did not show infiltration by the adrenal carcinoma. Therefore, according to two of the most extensive AATT series [6, 7], the percentage of preservation of the ipsilateral kidney was 17.4% (4 preserved of 23 possible).

The role of chemotherapy is controversial. Although various authors recommend early postsurgical regimens with mitotane [4], only partial responses have been described without an impact on survival [9]. The best results have been achieved with the combination of etoposide, cisplatin, and doxorubicin associated with mitotane, with response rates varying between 10% and 55% [10]. There are patients who respond well to the combination of surgery and chemotherapy, with a disease-free survival of 27.6% between 12 and 80 months after the end of chemotherapy [4].

## 4. Conclusion

We present a case of adrenalectomy and excision of a tumour thrombus preserving the ipsilateral kidney. There are objective data on patients with a good response to the combination of surgery and chemotherapy, and this justifies an attempt to preserve the renal function, despite the complex vascular approach. Consequently, prospective studies with a sufficient number of patients are necessary.
